# Testicular desmoplastic small round cell tumor: a case report and review of literature

**DOI:** 10.1186/1477-7819-12-227

**Published:** 2014-07-18

**Authors:** Gui-Ming Zhang, Yao Zhu, Hua-Lei Gan, Ding-Wei Ye

**Affiliations:** 1 Department of Urology, Fudan University Shanghai Cancer Center, No.270, Dongan Rd, Shanghai 200032, China; 2 Department of Oncology, Shanghai Medical College, Fudan University, Shanghai, China; 3 Department of Pathology, Fudan University Shanghai Cancer Center, Shanghai, China

**Keywords:** Desmoplastic small round cell tumor, Testis, Immunohistochemistry, Chemotherapy

## Abstract

**Background:**

Desmoplastic small round cell tumor (DSRCT) is an uncommon and highly aggressive malignancy with undetermined histogenesis and poor prognosis. To date, no case of testicular DSRCT has been reported in the literature.

**Case:**

A 42-year-old Chinese man presented with painless swelling of his left testis and a painless palpable nodule in his left inguinal region. Computed tomography showed a solid mass in the left testis and multiple metastases in the body. Laboratory tests gave no abnormal results. Left radical orchiectomy was performed, and histopathological and molecular pathological examination showed typical features of DSRCT. Six cycles of chemotherapy were administrated after the operation, leading to partial remission. Postoperative 9-month follow-up indicated no progression.

## Background

Desmoplastic small round cell tumor (DSRCT) is an extremely rare malignancy with undetermined histogenic origin. To date, about 300 cases have been reported in the literature, since it was first described as a mesenchymal entity with distinct clinicopathological features by Gerald and Rosai in 1989 [1,2]. DSRCT is prone to develop in adolescents and young adults, and the incidence in males is more than three times that in females [3]. The majority of DSRCTs originate from the abdominal cavity and pelvis, thus the retroperitoneum, omentum, and mesentery are often involved, in addition to multiple peritoneal tumors [4]. Although rarer, invasion of extraperitoneal sites, such as the central nervous system, nasal sinus, lung, bone and soft tissues, kidney, ovary and paratesticular region, has been documented [5-11]. However, no DSRCT derived from testis has been reported to date. Herein, we report a case of solid neoplasm in testis that was confirmed pathologically to be testicular DSRCT.

## Case presentation

**Figure 1:**
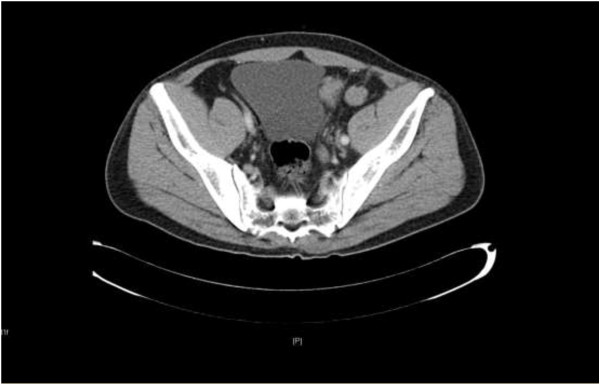
Computed tomography showed multiple lesions in left inguinal region, and near-bilateral iliac vessels, suggestive of multiple metastases.

A 42-year-old Chinese man was admitted to the Department of Urology at our hospital, with an 8-month history of painless swelling of his left testis and a 6-month history of a painless palpable nodule in his left inguinal region. He had no relevant personal or family history of malignancy.On physical examination, an egg-size solid lump was found in the left testis, and a palpable, slightly mobile, solid nodule, 2 × 3 cm in size, in the left inguinal region. Laboratory tests, including α-fetoprotein, β-human chorionic gonadotropin, and lactate dehydrogenase, gave no abnormal results. Computed tomography (CT) showed a confined solid mass, 2 × 3 cm in size with homogenous attenuation in the left testis, and multiple lesions in the right diaphragmatic crus, retroperitoneal region, and peritoneal and pelvic cavity, in the left inguinal region, and in the near-bilateral iliac vessels, suggestive of multiple metastases (Figure 1). In addition, the patient’s left renal pelvis and ureter were dilated.

**Figure 2:**
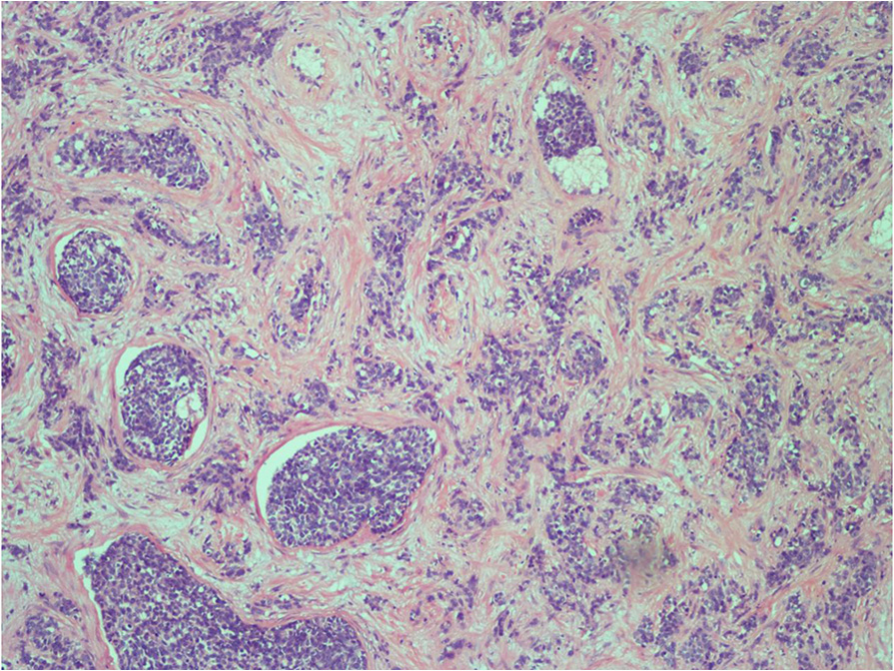
Well-defined nests of small round blue cells separated by abundant desmoplastic stroma (hematoxylin and eosin stain; magnification × 100).

**Figure 3:**
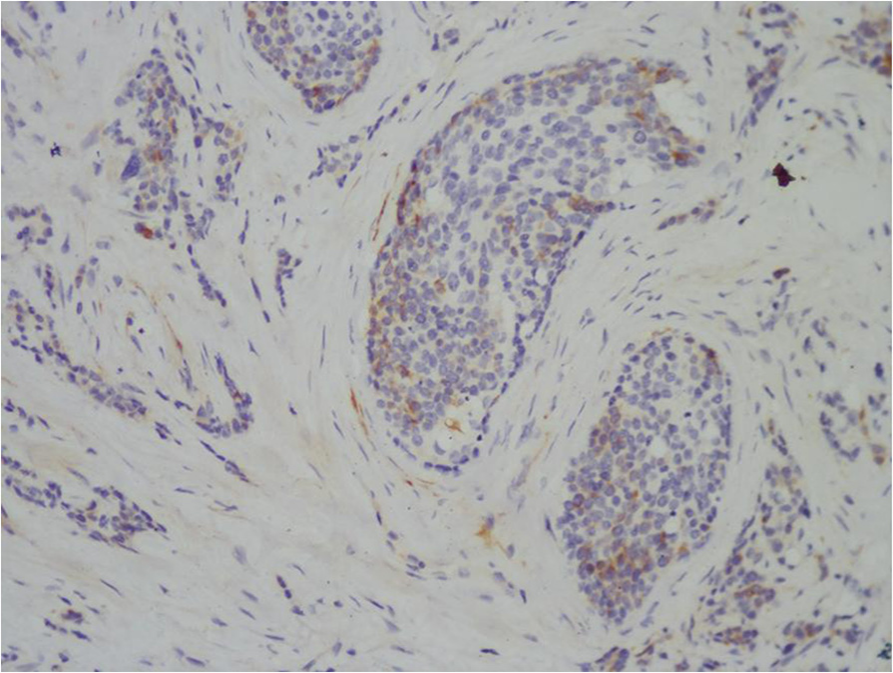
**Desmin was positive for immunohistochemistry.** Magnification × 200.

The preoperative diagnosis was left testicular tumor with multiple metastases. Left radical orchiectomy was carried out, and the cut surface of the tumor showed fish-like changes. Histopathological examination of the resected specimen revealed a malignant tumor composed of well-defined nests of small round blue cells, which were separated by abundant desmoplastic stroma (Figure 2). The spermatic cord and epididymis were also involved. Immunohistochemical evaluation exhibited the following: Cytokeratin Pan (+), Epithelial Membrane Antigen (+),Cytokeratin 7(−), vimentin (+), desmin (+) (Figure 3), Inhibin (−/+), Chromogranin A (weakly +), synaptophysin (+/−), CD56 (−), Placental ALK. Phosphatase (−/+),Wilm’s Tumor-1 (WT-1) protein (−), Myogenin (−/+), MyoD1 (−) and Neuron-specific enolase (+). Molecular evidence of t(11;22) (p13;q12) was detected by fluorescent in situ hybridization (FISH). Based on the abovementioned evidence, a diagnosis of testicular DSRCT was confirmed (pT_3_ N_2_ M_1_ S_0_).

**Figure 4:**
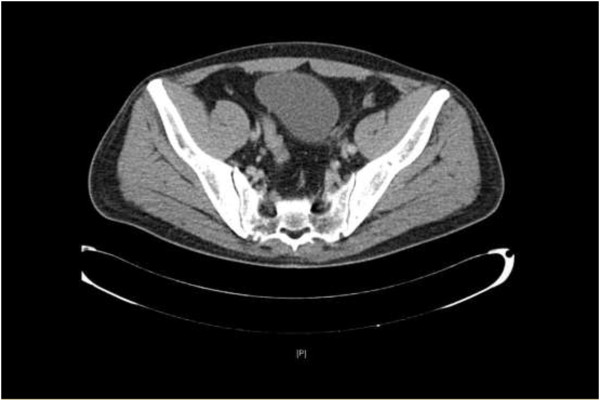
Computed tomography showed shrunken lesions in left inguinal region, and near-bilateral iliac vessels in the ninth postoperative month.

The patient was discharged on the fifth postoperative day in a good condition. In the third postoperative week, he was readmitted to the Department of Oncology at our hospital and received the VAC multi-agent systemic chemotherapy regimen consisting of vincristine (2 mg, day 1), adriamycin (75 mg/m^2^, day 1) and cyclophosphamide (1.2 g/m^2^, day 1). After six cycles of chemotherapy, the patient was appraised as being in partial remission. Postoperative 9-month follow-up indicated no progression as measured by CT assessment (Figure 4).

## Discussion

To our knowledge, this is the first case with testicular DSRCT documented in the literature. DSRCT has a male predominance and occurs most frequently in the second to third decades of life [3]. As it can have delitescent onset without apparent symptoms and is highly aggressive, DSRCT is often diagnosed at a relatively advanced stage. Furthermore, owing to the paucity of this tumor, there is no optimal treatment strategy and favorable long-term survival have not been achieved.

The diagnosis of DSRCT remains challenging. Because most DSRCTs are found in the abdominal cavity, some non-specific gastrointestinal symptoms (such as nausea, vomiting, abdominal distension and pain) and a palpable abdominal mass, are the main problems leading to patients visiting a doctor. If present in extraperitoneal sites, DSRCT can cause specific symptoms, mostly owing to expansion and compression of the tumor. Weight loss, acratia, ascites, and cachexia may occur when there is tumor dissemination or extensive lymphogenous and hematological metastases. In our case, the patient was in good general condition when he visited his doctor, but multiple metastases had already developed. The tumor features, such as non-specific manifestations and high aggression, meant that the patient was already experiencing late-stage disease at his first visit.

Imaging methods can help to detect DSRCT, although there are no typical characteristics. CT scans show homogenous low attenuation with modest uniform enhancement on contrast-enhanced CT in small masses, while central low attenuation with modest heterogeneous enhancement on post-contrast CT, suggestive of central hemorrhage or necrosis, may be observed in cases of larger masses [12]. In addition, calcification is relatively common. Ultrasonography and magnetic resonance imaging also reveal one or multiple nodules ‘studding‘ the peritoneal cavity or other locations [13]. Positron emission tomography (PET)/CT is recommended as an effective evaluation of tumor staging [14]. In a retrospective study, Arora *et al*. evaluated the role of fluorodeoxyglucose PET/CT for diagnosis of DSRCT, and found an accurate detection of 97.4% of all DSRCTs, with sensitivity, specificity, positive predictive value, and negative predictive value of 96.1%, 98.6%, 98.0%, and 97.1%, respectively [12]. Recently, fine-needle aspiration cytology has been tried, and found to provide another effective approach for early diagnosis and chemotherapeutic response assessment of DSRCT [15].

Microscopically, DSRCT consists of well-defined nests of small round blue cells with hyperchromatic nuclei and indistinct nucleoli, which are separated by affluent desmoplastic stroma. Peripheral palisading of tumor cells and rosette-like structures may be observed [1,16]. Immunohistochemistry demonstrates divergent differentiation of DSRCT, which can be used as a definite diagnostic marker for DSRCT. The tumor stains positivelhy for several epithelial (e.g. keratin), mesenchymal (e.g. vimentin), myogenic (e.g. desmin), and neural (e.g. CD56) markers, thus desmin and vimentin together produce a unique staining pattern, namely the punctuate and perinuclear cytoplasmic positivity, which is a notable trait of DSRCT [16]. C19, an antibody to the C-terminal region of the WT1 protein, has been proven useful for differentiating DSRCT from Ewing sarcoma/primitive neuroectodermal tumor (EWS/PNET) when genetic information is unavailable [17]. The presence of the specific cytogenetic abnormality t(11;22)(p13;q12) is observed in more than 90% of all patients [18], which eventually promotes uncontrolled proliferation of DSRCT cells [19]. Several staging systems have been suggested for classification of peritoneal malignancies [20], but no validated staging system has been proposed for DSRCT. Recently, a new staging system based on the Peritoneal Cancer Index was proposed, by which comparison of DSRCT outcomes may be applied, although this was carried out with only a small cohort of patients and needs further validation [21]. In our case, we used the staging system established by UICC [22] in 2002 for testicular tumor, because no other available staging systems were suitable for this rare case.

In general, DSRCT must be distinguished from other small round cell tumors, such as EWS/PNET, neuroblastoma, and lymphoma. Similarly to DSRCT, EWS/PNET is also composed of small round cells in sheets or nests. Typically, EWS/PNET is positive for CD99 and vimentin, and negative for cytokeratins and myogenic markers. Different from DSRCT, which has a translocation of t (11;22)(p13;q12), EWS/PNET has a characteristic reciprocal chromosomal translocation of t(11;22)(q24;q12) [16]. Neuroblastoma often occurs in children. Tumor cells are negative for CK, WT1 and desmin, and the stroma is rich in nerve fiber network, with ganglion cell differentiation commonly observed [23]. High-grade lymphoma may share similar histologic features with DSRCT, however, lymphomas often display a diffuse growth pattern. Positive lymphoid markers and negative epithelial and myogenic markers can help with the differential diagnosis [24].

Currently, no consensus has been made as to the treatment protocols for DSRCT [25]. Debulking surgery, radiotherapy, and chemotherapy are possible therapeutic strategies, although none of them appears to have better efficacy than the others. Debulking surgery is still the main approach of DSRCT management. In a retrospective study, a 3-year survival rate of 58% was reported for patients who received debulking surgery, whereas no patients in the non-surgical group survived for more than 3 years [26]. Although DSRCT is somewhat sensitive to chemotherapy and radiotherapy, durable remissions remain rare [27]. Chemotherapeutic agents commonly administrated for DSRCT include doxorubicin, cyclophosphamide, vincristine, ifosfamide and etoposide. Hyperthermic intraperitoneal chemotherapy (HIPEC) has been performed in some institutions [28,29], and the 3-year survival in patients receiving debulking surgery with HIPEC was 71%, compared with 62% in patients who underwent surgery alone; however this did not reach statistical significance [17]. Radiotherapy is helpful in prolonging life but does not promote long-term survival [1].

Generally speaking, the prognosis of DSRCT is poor. A median progression-free survival of 2.6 years was reported by Schwarz *et al*., and another study group reported that 71% of patients died within a mean of 25.2 months [30,31]. Because no standard therapeutic strategy has been recommended, we administrated the VAC protocol to our patient after debulking surgery, following the treatment for sarcoma, and there was no progression appeared at the postoperative 9-month follow-up. However, the patient requires further observation due to the follow-up time.

## Conclusions

DSRCT is a relatively uncommon and highly aggressive tumor, with poor prognosis. The diagnosis is usually established by immunohistochemistry. To date, no standard treatment protocol has been proposed. More investigations are warranted to define the optimal therapeutic strategy.

## Consent

Written informed consent was obtained from the patient for publication of this case report and any accompanying images. A copy of the written consent is available for review by the Editor-in-Chief of this journal.

## Abbreviations

DSRCT: Desmoplastic small round cell tumor; EWS: Ewing sarcoma; CT: Computed tomography; FISH: Fluorescent *in situ* hybridization; HIPEC: Hyperthermic intraperitoneal chemotherapy; PET: Positron emission tomography; PNET: primitive neuroectodermal tumor; WT-1: Wilm’s tumor1.

## Competing interests

The authors declare that they have no competing interests.

## Authors’ contributions

GMZ and YZ conceived of the concept, and participated in drafting the manuscript. HLG reviewed the pathological slides and revised the manuscript. DWY supervised the project and revised the manuscript. All the authors read and approved the final version and agreed to publish the manuscript.
